# Editorial: Danger Signals Triggering Immune Response and Inflammation

**DOI:** 10.3389/fimmu.2017.00979

**Published:** 2017-08-11

**Authors:** Abdulraouf Ramadan, Walter G. Land, Sophie Paczesny

**Affiliations:** ^1^Department of Pediatrics, Indiana University School of Medicine, Indianapolis, IN, United States; ^2^Department of Microbiology Immunology, Indiana University School of Medicine, Indianapolis, IN, United States; ^3^Melvin and Bren Simon Cancer Center, Indiana University School of Medicine, Indianapolis, IN, United States; ^4^German Academy of Transplantation Medicine, Munich, Germany; ^5^Molecular ImmunoRheumatology, INSERM UMR_S1109, Laboratory of Excellence Transplantex, Faculty of Medicine, University of Strasbourg, Strasbourg, France

**Keywords:** damage-associated molecular pattern molecules, pathogen-associated molecular patterns, inflammation, toll-like receptors, inflammatory diseases

**Editorial on the Research Topic**

**Danger Signals Triggering Immune Response and Inflammation**

The immune system detects “danger” through a series of what we call pathogen-associated molecular patterns (PAMPs) or damage-associated molecular pattern molecules (DAMPs), working in concert with both positive and negative signals derived from other tissues. PAMPs are molecules associated with groups of pathogens that are small molecular motifs conserved within a class of microbes. They are recognized by toll-like receptors (TLRs) and other pattern-recognition receptors. A vast array of different types of molecules can serve as PAMPs, including glycans and glycoconjugates. Bacterial lipopolysaccharides (LPSs), endotoxins found on the cell membranes of Gram-negative bacteria, are considered to be the prototypical class of PAMPs. LPSs are specifically recognized by TLR4, a recognition receptor of the innate immune system. Other PAMPs include bacterial flagellin (recognized by TLR5), lipoteichoic acid from Gram-positive bacteria, peptidoglycan, and nucleic acid variants normally associated with viruses, such as double-stranded RNA, recognized by TLR3 or unmethylated CpG motifs, recognized by TLR9. DAMPs, also known as alarmins, are molecules released by stressed cells undergoing necrosis that act as endogenous danger signals to promote and exacerbate the immune and inflammatory response. DAMPs vary greatly depending on the type of cell (epithelial, mesenchymal, etc.) and injured tissue. Some endogenous danger signals include heat-shock proteins, high-mobility group box 1 (HMGB1), reactive oxygen intermediates, and extracellular matrix breakdown products such as hyaluronan fragments, neuromediators, and cytokines including the interferons. Non-protein DAMPs include ATP, uric acid, heparin sulfate, and DNA. Furthermore, accumulating evidence supports correlation between alarmins and changes in the microbiome. Increased serum or plasma levels of these DAMPs have been associated with many inflammatory diseases, including gastric and intestinal inflammatory diseases, graft-versus-host disease (GVHD), sepsis and multiple organ failure, allergies particularly in the lungs, atherosclerosis, age-associated insulin resistance, arthritis, lupus, neuroinflammation/degeneration, and more recently in tumors, which is particularly interesting with the emergence of immunotherapies. Therapeutic strategies are being developed to modulate the expression of these DAMPs for the treatment of these diseases.

A vast number of reviews have already been published in this area; thus, in an effort to not duplicate what has already been written, we will focus on recent discoveries particularly in disease models that are epidemic in Western society: intestinal chronic inflammatory diseases including GVHD and its relationship with the microbiome, chronic infectious diseases, allergies, autoimmune diseases, neuroinflammation, and cancers. We will also focus on the basic cellular roles of macrophages, T cells, and B cells.

This *research topic* brings together 16 articles that provide novel insights into the mechanisms of action of DAMPs/alarmins and their regulation and subsequent immunologically driven responses. The take-home messages from these 16 studies are summarized below.

Two articles focused on the basic mechanisms of activation of macrophages and B cells by TLRs. Chu et al. showed in an original research article that the Fab fragment of a human anti-Siglec-9 monoclonal antibody suppresses LPS-induced inflammatory responses in human macrophages, which has important therapeutic consequences for sepsis management. The mini-review by Suthers and Sarantopoulos explored the cross talk between TLR7/TLR9 and BCR signaling, which they suggest induces dangerous B cells. Although underexplored, it is now clear that a balance between TLR7 and TLR9 is pivotal in the development of B-cell autoreactivity, and one disease model to study this further is chronic GVHD, as the microenvironment after allogeneic hematopoietic stem cell transplantation contains large amounts of microbial-derived nucleic acids and B-cell-activating factor.

Five articles included in this *research topic* investigated the roles of PAMPs and DAMPs in the development of intestinal inflammation including acute GVHD. Pellegrini et al. reviewed how the canonical and non-canonical activation of the NLR family pyrin domain containing 3 (NLRP3) inflammasome regulates tolerance and inflammation in the intestine. Indeed, NLRP3 has a dual role in the pathogenesis of bowel inflammation. In some studies, NLRP3 has regulatory and reparative roles in the immune homeostasis and maintenance of the epithelial barrier integrity, whereas in others, overactivation of NLRP3 contributes to interruption of the intestinal immune balance. Lin et al. published an original article on how *Helicobacter pylori*, which infects more than half of the human population worldwide, activates HMGB1 expression, and recruits RAGE into lipids rafts to promote inflammation in gastric epithelial cells. In this translational work, the authors found that HMGB1 and RAGE expression levels are significantly greater in *H. pylori*-infected cells than in uninfected gastric cells. Blocking HMGB1 by a neutralizing antibody can abrogate *H. pylori*-elicited RAGE signaling by reducing nuclear factor (NF)-κB activation and interleukin (IL)-8 production. The current understanding and future perspectives of danger signals in GVHD were reviewed by Toubai et al. One interesting new finding in this field is the role of the HMGB1 receptor (Siglec-G)/CD24 axis in controlling the severity of GVHD (1). The mini-review by Apostolova and Zeiser summarizes the role of purine metabolites, particularly ATP/ectonucleotidases as novel DAMPs, in the development of acute GVHD. These interactions are influenced by the intestinal microbiome, which has been particularly well explored. Slingerland et al. reviewed the role of the microbiome in intestinal diseases and other diseases such as circulatory, integumentary, musculoskeletal, respiratory, neuromuscular, and systemic conditions, focusing on clinical evidence and one potential therapeutic intervention. Two original papers looked at danger signals in relation to autoimmunity development. Peng et al. showed increases in TLR activity and TLR ligands in patients with autoimmune thyroid diseases. Using peripheral blood mononuclear cells from 30 healthy controls, 36 patients with untreated Hashimoto’s thyroiditis, and 30 patients with newly onset Graves’ disease, they showed that TLR2, TLR3, and TLR9 expression and activation are increased in patients with autoimmune thyroid diseases, suggesting a role for TLRs in the pathogenesis. Yun et al. showed that the HMGB1–CXCL12 complex promotes T-cell infiltration in chronic experimental autoimmune uveitis. They demonstrated that at a very early stage of intraocular inflammation initiated by uveitogenic autoreactive T cells, synergism between HMGB1 and CXCL12 is crucial for the infiltration of inflammatory cells and that the induction of experimental autoimmune uveitis was significantly inhibited by a CXCR4 antagonist, AMD3100.

Three papers explored the role of danger signals in the brain. Terrando et al. showed that HMGB1 is rapidly released after tissue trauma and its neutralization prevents postoperative neurocognitive dysfunction in a model of aged rats. Indeed, postoperative neurocognitive disorders are common complications in elderly patients following surgery or critical illness, and these findings offer a better understanding of the neurocognitive dysfunction and therapeutic options. Lin et al. studied another neuroprotective effect through the activation of the cannabinoid receptor-2 with a selective agonist, JWH133, and they showed the protection to be due to suppressed neuroinflammation and upregulated expression of microglial macrophage M2-associated markers in an intracerebral hemorrhage model. Finally, Wilkins et al. reviewed the role of mitochondria-derived DAMPs in neurodegeneration. The roles of neuroinflammation in neurodegenerative diseases including Alzheimer’s disease (AD), Parkinson’s disease, and amyotrophic lateral sclerosis are increasingly appreciated, but the field is still in its infancy. Initial interest in neuroinflammation as a causative factor in AD was triggered by the reduced risk for AD in long-term non-steroidal anti-inflammatory drug users, which was later linked to the apolipoprotein E ε4 allele (2). Furthermore, genome-wide association studies identified a triggering receptor variant expressed on myeloid cells 2 (TREM2) as a causative factor for AD (3). Since these initial studies were published, much progress has been made to clarify how mitochondrial dysfunction plays a fundamental role, as elegantly reviewed by the group of Russell Swerdlow, a pioneer in the field (Wilkins et al.).

Two studies investigated the importance of immune homeostasis disruption in lung diseases. Chen et al. demonstrated that a novel subset of IL-10-producing CD1d^hi^CD5^+^ regulatory B cells modulates immune homeostasis in patients with silicosis, a condition of chronic inflammation and fibrosis of the lung. The study reported by Lin et al. showed that LPS can attenuate proallergic cytokines such as thymic stromal lymphopoietin (TSLP) and IL-33 in respiratory epithelial cells stimulated with polyI:C and human parechovirus. This work supports the “hygiene hypothesis,” which claims that childhood exposure to environmental microbial products is inversely related to the incidence of allergic diseases in later life. It also suggests that in addition to therapeutic targeting of TSLP and IL-33, local application of non-pathogenic LPS may be a rational strategy to prevent allergies.

Interestingly, one of the mediators recently identified in the pathogenesis of allergies is IL-33. Undeniably, the serum stimulation-2 (ST2)/IL-33 axis has been found to be rooted in the pathogenesis of an increasing number of diseases, and this is reviewed in a manuscript published by Griesenauer and Paczesny. Briefly, ST2, the IL-33 receptor, exists in two forms as splice variants: a soluble form (sST2), which acts as a decoy receptor, sequesters free IL-33, and does not signal, and a membrane-bound form (ST2), which activates the MyD88/NF-κB signaling pathway to enhance mast cell, Th2, regulatory T cell, and innate lymphoid cell type 2 functions. Plasma/serum levels of sST2 are increased in patients with active inflammatory bowel disease (4), cardiac diseases (5), acute cardiac allograft rejection (6), and GVHD (7–14). The review also details the immune cells that express ST2 on their surface or secrete sST2 as well as the relevant signaling mechanisms. The ST2/IL-33 axis has recently been shown to be a potential novel checkpoint in the development of tumors, as reviewed by Wasmer and Krebs. Indeed, recent findings have shown a role of IL-33 in several cancers where it may exert multiple functions. The role of the ST2/IL-33 axis has been particularly well studied in colorectal cancer (15) and myeloproliferative neoplasms (16). Importantly, IL-33 could be used as potential tumor biomarker or therapeutic target.

In summary, the reviews and original articles collected for this research topic of *Frontiers in Immunology* convey to readers the multiplicity and implications of PAMPs/DAMPs/alarmins and their regulators in the development of inflammatory diseases. Importantly, learning from these mechanisms of action, each group of investigators has proposed novel targeted treatments.

## Author Contributions

All authors wrote and approved the manuscript.

## Conflict of Interest Statement

The authors declare that the research was conducted in the absence of any commercial or financial relationships that could be construed as a potential conflict of interest.
